# What *N* Is N-ough for MRI-Based Animal Neuroimaging?

**DOI:** 10.1523/ENEURO.0531-23.2024

**Published:** 2024-03-06

**Authors:** Joanes Grandjean, Evelyn M. R. Lake, Marco Pagani, Francesca Mandino

**Affiliations:** ^1^Donders Institute for Brain, Cognition, and Behaviour, Nijmegen 6500HB, The Netherlands; ^2^Department for Medical Imaging, Radboud University Medical Center, Nijmegen 6500HB, The Netherlands; ^3^Departments of Radiology and Biomedical Imaging, New Haven, Connecticut 06519; ^4^Biomedical Engineering, Yale School of Medicine, New Haven, Connecticut 06519; ^5^Functional Neuroimaging Laboratory, Center for Neuroscience and Cognitive Systems, Istituto Italiano di Tecnologia, Rovereto 38068, Italy; ^6^IMT School for Advanced Studies, Lucca 55100, Italy

**Keywords:** animal neuroimaging, fMRI, rodent, sample size

## Abstract

Fueled by the recent and controversial brain-wide association studies in humans, the animal neuroimaging community has also begun questioning whether using larger sample sizes is necessary for ethical and effective scientific progress. In this opinion piece, we illustrate two opposing views on sample size extremes in MRI-based animal neuroimaging.

Picture this. You are a senior PhD candidate delivering the concluding statements of your talk. The audience is a diverse group of neuroimagers who have assembled from across the globe to share their science. Some work in human neuroimaging, while others work with animals. You are in the latter group which makes up the minority of the attendees. As such, you have spent precious minutes during your presentation outlining the culmination of years spent in the lab painstakingly troubleshooting your experimental design before moving on to a summary of your findings. A few short slides, that fail to do the amount of work and perseverance justice. Your talk ends, the audience applauds, and now, it is time for questions. Inevitably, someone asks: “I noticed that you only have 20 animals in your experiment (10 per group). Given what we now know about small “N” – don't you need a lot more data?” Your heart sinks because as of this moment, the conversation is no longer about your thesis, it is now about one of the looming elephants in the room—“what N is N-ough for MRI-based animal neuroimaging?”—and there is no “correct” answer.

Now, picture the moment (before you answer) frozen in time. Let us imagine a symbolic angel and devil appear on your shoulders—only, there is no “right” and “wrong” side—just two differing perspectives. On your left, Joanes appears ready to argue for large (*N* = 1,000) sample sizes in MRI-based animal neuroimaging. On your right, Francesca appears prepared to counter these arguments and defend smaller *N* studies. What follows is a lively debate.

***Joanes’ summary:*** *Effects in biology are rarely large. This is true for human neuroimaging studies, but also for animal-based research. We need to adapt our study designs to properly investigate the medium and small effects that are relevant to biomedical and neuroimaging sciences.*

## Large *N* Is Becoming the Norm for MRI-Based Human Neuroimaging

The “*Why Most Published Research Findings Are False*” essay is a dire constatation for modern biomedical science (Ioannidis, 2005). The notion that we are wasting our resources (funding, person-power, time, and attention) on studies that will not replicate or simply add to the scientific background noise is unbearable. The ethics of it is far worse when considering the number of animals used in biomedical research. We must maximize the utility of every animal used, yet I think we are failing in that regard. The “*N* = 1,000 participants’ recommendation for brain-wide association studies in humans position paper” was announced with fracas and divided the human MRI community (Marek et al., 2022). The authors argued that most brain-wide associations found in large neuroimaging datasets have medium to small effects and, therefore, that future studies on the topic should consider plausible effect sizes when estimating their required group size and adapt accordingly. The risks of ignoring this cautionary warning are underpowered studies prone to false positives and negatives that contribute to the scientific background noise if little else.

## Are the Considerations for Large *N* Different for Animal Studies?

I think not. The median estimated effect size in animal behavior studies (of *N* = 479 studies surveyed) is Hedge's *g* ∼ 0.5, corresponding to a medium effect according to Cohen's 1998 interpretation guidelines (Bonapersona et al., 2021). This implies that 50% of the studies in that pool would need sample sizes of >100 per group and >1,000 for the bottom 25% of the animal behavioral studies with the smallest effect sizes. This is a far cry from the median sample size *N*  = ∼ 10 that we traditionally use. These results support the notion that sample sizes in animal research do not match the effect sizes under investigation.

But surely transgenic rodents have larger effects? On the surface, this argument seems plausible. Compared with studies in humans where genetic differences between individuals are slight (yet numerous), we use isogenic animals with large differences (knock-in or knock-out) in one or a few genes. This should amplify the biological signal while mitigating noise from the uncontrolled environment and genetic makeup. In practice, this argument is more nuanced. We can learn from the (very) few studies that perform meta-analyses on animal data, the gold standard in evidence-based research. For instance, the SERT^−/−^ rodent model of depression/anxiety with a knock-out for the serotonin reuptake transporter has an aggregated large effect size for defensive/anxiety-related behavior relative to wild-type controls (*g* ∼ 0.88 and 95% confidence interval [0.65, 1.1], based on 13 studies; Mohammad et al., 2016). However, when accounting for publication biases, for which there was evidence, the effect size was corrected to a medium effect (*g* ∼ 0.57 [0.29, 0.86]). Hence, we cannot exclude that knock-out animals, and transgenics in general, may have more modest effects than originally thought and that publication biases currently skew these effects.

## Are Effects Larger in MRI-Based Animal Neuroimaging than Behavioral Studies?

MRI-based animal studies are also performed with relatively small sample sizes (median *N*  = ∼ 15 for rat studies; Mandino et al., 2019). I would be surprised if effect sizes, on average, were any larger than those described for behavioral studies, especially for functional parameters at the detection limit and prone to measurement artifacts. Neuroimaging studies, however, usually estimate more parameters (e.g., functional connectivity matrices with 100 × 100 parameters) than behavioral studies. This leads to multiple hypothesis testing and adds extra requirements for multiple test corrections, which further reduces statistical power relative to behavioral studies. Moreover, neuroimaging studies have greater analytical flexibility, which makes them more vulnerable to post hoc selective analyses and indirectly amplifies effect size via publication biases (Carp, 2012). All these factors contribute to making animal neuroimaging prone to effect size overestimation.

The advent of standardized acquisition protocols and processing software for animal neuroimaging promises to mitigate some of these issues (Grandjean et al., 2023). They can limit analytical flexibility by providing default processing parameters (Desrosiers-Gregoire et al., 2022). At the same time, standardized acquisition promises to ease meta-analyses by reducing differences between centers and studies. The current protocols and pipelines we designed are also amenable to being scaled up, a prerequisite for big *N* datasets. Yet, until these measures pick up in our community, the dire reality is that many of our animal neuroimaging studies are likely underpowered and prone to spurious outcomes.

## Small Effects Are Not Bad. It Is Our Study Designs That Are Inadequate

There is building evidence from both human and animal research that the effects in biology are more modest than initially reported. This is not inherently bad. Antidepressants have a small effect (*d* ∼ 0.3 for classical antidepressant vs placebo; Cipriani et al., 2018), and so do antiamyloid therapies (*d* ∼ 0.23 for antiamyloid interventions vs placebo; Mintun et al., 2021; Goldberg et al., 2023). This does not undermine their clinical utilities as the first line of defense against depression and a new hope for patients with Alzheimer's disease, respectively. I think it is time to accept that we are facing challenges in biomedical research that the traditional ways of doing our experiments cannot handle. We have the means to pool our resources, either via the collection of large datasets within single centers, as the Allen Institute for Brain Science is doing (Lein et al., 2007; Oh et al., 2014), or smaller datasets amassed from multiple centers as the International Brain Laboratory (International Brain Laboratory, 2017) and our multicenter rodent neuroimaging studies are doing (Grandjean et al., 2023, 2020). It is time to face the biological realities and acquire data that can make a true scientific difference.

***Francesca’s summary:*** *For animal neuroimaging, increasing N by a factor of one hundred is an intractably expensive option – ethically and otherwise – given how we fund and conduct our research. It also assumes that what seems to be necessary for human research is also necessary for animal research – but these are fundamentally different…animals. Animal neuroimaging is a much nimbler field with better alternatives than the heavy-handed strategy of a blanket increase in the number of subjects. Increasing N to address challenges in animal neuroimaging fails to play to the strengths of the field and to recognize the differences between the realities of animal and human neuroimaging research.*

## What Are We Trying to Accomplish?

The goal of both human and MRI-based animal neuroimaging is to understand the *human* brain and/or devise new methods and treatments relevant to humans. In the scenario where we are successful, understanding the brain means we can turn measurement into action at the level of the individual. This is the holy grail of personalized medicine. If we need 1,000 measurements to have the sensitivity to detect an effect, perhaps we need to consider improving our measurements (rather than simply making more of them). This is easier to accomplish, first, in animal research where we have much greater control and flexibility.

## The Unparalleled Power (and Dimensionality) of Animal Research

In some ways, using animals is a (big and necessary) compromise (their brains are an evolutionarily simplified version of our own). Yet, research on animals gives us unprecedented neurobiological access, tight control of genetic and environmental factors, as well as the ability to study the lifespan on a tractable timescale (e.g., mice live for ∼24 months). We can also target specific aspects of a pathology at first, and then build on this knowledge base (in effect titrating the complexity of what we are studying). Consider Alzheimer's disease as an example. There are a variety of animal models that mimic various phenotypes of the disease (e.g., APP/PS1 mice model overt β-amyloid plaque accumulation, whereas 3xTgAD mice model both plaque accumulation and tauopathy; Oddo et al., 2003; Jullienne et al., 2022; Mandino et al., 2022;Yokoyama et al., 2022). Many of the dimensions in animal research have no counterpart in humans. These are immensely powerful abilities, but they also increase the number of possible experiments. With more options and fewer researchers, we are only just scratching the surface of what can be accomplished using animal neuroimaging. Better ways of harmonizing how we acquire and process these data are also only just emerging and evolving at a fast pace. It would be constraining the diversity of animal experimentation at this early stage just to boost *N*. Obtaining large numbers of homogenized data necessitates reducing the complexity (and creativity) of the experiment (Williams, 2010). At this early juncture, I think we stand to gain more from animal research if we continue to invest in expanding the breadth of what we can do.

## Humans Are Similar, Mice Are (Almost) Identical: Do We Really Understand the Sources of Heterogeneity in Our Neuroimaging Data?

Humans, although they share 99.6% of their DNA, are extremely different from one another (Maxwell et al., 2019). They also live under highly variable conditions (e.g., from noisy suburban scenes to peaceful countryside) and experience a human lifetime's worth of exposure to change (e.g., global warming). Research animals, for example the classic C57BL6 mice, are carefully engineered to be as identical as possible (although variations will always be present, e.g., at the epigenetic level) and live under tightly controlled conditions (same light/dark cycles, same humidity, every day, every year). Yet, lab animals still develop diverse behaviors (Freund et al., 2013). In the face of this persistent heterogeneity (despite our best efforts), we should be humbled by how exponentially different “non-identical human beings living in very non-identical environments across the globe” truly are.

## Increasing *N* Is Not Our Only Option for Improving Our Science

Demanding a larger number of animals to address the problem of publication bias is an appalling suggestion. The lives of the animals we work with should not be forfeited to fix our problems with publishing. Funding and resources for experimental work are precious and finite. Publishing null or negative results has (almost) no cost and contributes to scientific progress (as much as—if not *more*, in the current climate—as positive results). The ethical course of action is to work on changing our cultural publication biases—not increasing the number of animals to compensate for our egos. We should accept null or negative results, and pure replication studies, as equal contributors to the scientific discourse *before* we consider scaling up our numbers. Rigorous statistical analyses, corrections for multiple comparisons, and better reporting practices must also come first.

## What If the Effect Size *Is* Too Small to Detect with a Small *N*? Is *N* = 1,000 Ethical in Animal Research?

No. Working with animals is a privilege. If the biological question necessitates *N* = 1,000 to detect an effect, then either the question is not properly framed or the means of investigation being used are not sufficiently developed. In human research, we work with imaging modalities that are noninvasive and easily tolerated. Participants give consent and are offered compensation (e.g., monetary payment). They then continue with their lives. In other words, the best interests and comfort of our participants are the priority. This makes the “ethical cost” of conducting *N* = 1,000 imaging sessions in humans *negligible*. The landscape in animal research is completely different.

Animals are not capable of giving consent. In the pursuit of answering any research question, the life of every animal used is forfeited. This fact leads to an ethical obligation to minimize the number of animals used for research. This ethos is captured well by the “Three Rs principle”: Replacement, Refinement, and Reduction (Russell, 1995; Hooijmans et al., 2010). (1) Explore alternatives to animals (e.g., in silico options). (2) Develop experimental procedures that minimize suffering and maximize the utility of each animal. (3) Use every means available to minimize the number of animals needed to answer a scientific question. The proposition that *N* = 1,000 is necessary to make the lives of those animals “well spent” is an ill-positioned argument. Instead, we need to ask what replacements and refinements will make *N* = 10 sufficient.

## *How* Animal Neuroimaging Data Are Obtained *Matters*

Neuroimaging data from humans is typically collected by technicians who are permanent staff. The protocols that are followed are highly standardized (especially when they are part of large *N* studies) and generally very easy to implement. Conversely, most neuroimaging data from animals are collected by trainees (students, or postdoctoral fellows) and junior researchers at critical points on their career paths. In part, this is because acquiring neuroimaging data from animals is far from trivial or standardized. The necessary skills easily encompass basic-to-complex animal care (e.g., breeding and genotyping), surgical manipulations (and post-op recovery), delivery of anesthesia, intubation/extubation and ventilation, animal training (e.g., for imaging awake subjects), maintaining animal physiology during imaging (e.g., body temperature, heart and breath rate, blood oxygen, or expired carbon dioxide), and more. Acquiring proficiency in these skills takes years of training and practice—most of which occurs within a small number of specialized research labs.

As the introduction tried to illustrate—this is an underappreciated bottleneck on the road to scaling general access to animal neuroimaging data and data volume. It is challenging to argue that it is in the best interest of those collecting the data to devote such substantial time and energy even for small *N* studies. There are very real and practical reasons why animal neuroimaging data is a scarce and valuable resource. These need to be part of the conversation when we talk about scaling our operations.

This argument only becomes more relevant when we consider the newest contributions being made by animal neuroimaging (e.g., multimodal methods like optogenetics, DREADDs, or WF-Ca^2+^ in combination with functional magnetic resonance imaging, e.g., Lake et al., 2020; Mandino et al., 2022; Rocchi et al., 2022). Pushing these frontiers demands even more expertise and time from an even more limited pool of scientists. Having one person perform the same procedure on 100 times the number of animals or a handful of experimenters (if you can find them) is impractical for the most promising and creative work being done. It is only tractable for the most basic experiments. It would be foolish to sacrifice this high level of quality for simple quantity.

## Are We Able/Ready to Handle Big Datasets?

Given the experimental flexibility, unique challenges, and a (relatively) small community of animal neuroimagers, it is unsurprising that our data acquisition methods and preprocessing strategies are far from standardized (Mandino et al., 2019, 2024; Grandjean et al., 2023, 2020). This reflects both some of the weaknesses and strengths of animal neuroimaging but also its newness. It is simply too early to push for *N* = 1,000 from an acquisition standpoint—or (arguably) a data infrastructure standpoint. Large *N* studies are only useful if there are resources that support equitable, safe, and smooth data storage and usage. Immense effort has gone into establishing these resources for human neuroimaging data (Van Essen et al., 2012, 2013). From this example, much has been learned about how best to do the same for animal neuroimaging (Gorgolewski et al., 2016; Buckser, 2021; Markiewicz et al., 2021; Desrosiers-Gregoire et al., 2022). However, there are some notable differences between these endeavors which will require more work (and investment) before substantial scaling can take off. One of the foremost challenges is adequately crediting the individuals who collect animal neuroimaging data (see preceding section). Further challenges lie in properly cataloging these incredibly diverse data (e.g., collating multiple modalities). Granted—these are not insurmountable—however, we are not there yet, and getting there will take a large shift in how we think about shared data and how we invest in supporting the development and maintenance of this infrastructure.

## What Should We Focus on, Instead?

When it comes to the future, we benefit most when we are mindful of our present landscape. Large *N* data is only one of many possibilities for our field, and it is a remarkably expensive one. In animal neuroimaging, there is a big push toward multimodal, cross-disciplinary, and cross-species approaches, especially in studying complex pathologies like Alzheimer's disease. In my opinion, we stand to gain more from leveraging the flexibility and controllability of studies on animals than boosting their number. We should invest in our strengths and continue to collect rich data using multiple complementary modalities. Promoting better scientific practices (e.g., publishing negative results) will accelerate our progress. Yet, our focus should be on uncovering the means to create new and more refined measurements (not on increasing the number of measurements that we know to be poor). An improved understanding of, and ability to work with, our data does not automatically come from having a lot of it. It is more likely to come from a better understanding of the neurobiological phenomena that they report on. Success should never be *N* = 1,000. It should be *N* = 1.

## End Debate—Scene Unfreezes—Time to Answer the Question

Spurred by recent brain-wide association studies in humans (Marek et al., 2022), the animal neuroimaging community has also begun reasoning on whether to use larger samples. In general, sample size has been based on dogma and feasibility. Now, the cost–benefit analysis of small versus large has taken on a more pragmatic and analytical dimension. From a statistical and interpretational standpoint, the advantages of large samples are hard to debate. You can do more with more confidence when you have more data.

A “wide” database composed of a large number of empirical observations (i.e., brain images) allows the robust investigation of a novel class of research questions of high relevance to neuroscience. The results of these efforts are epitomized by prominent human neuroimaging studies on interindividual variations across the lifespan (Bethlehem et al., 2022), reproducibility of brain–behavior associations (Marek et al., 2022), functional mapping of primary motor cortex (Gordon et al., 2023), and atypical functional connectivity in neuropsychiatric disorders (Di Martino et al., 2014). Importantly, the advent of data-sharing initiatives in rodents has recently allowed animal neuroimagers to compile sufficient data resources to rigorously quantify the reproducibility of mouse functional connectivity networks across labs (Grandjean et al., 2020) and develop a consensus protocol for rat neuroimaging (Grandjean et al., 2023). These are two pillars of rigor and reproducibility in animal research. Analogously, multicenter neuroimaging studies of nonhuman primates ({PRIMatE Data Exchange (PRIME-DE) Global Collaboration Workshop and Consortium, 2020) have contributed maps of evolutionarily conserved brain hierarchies (Xu et al., 2020). These discoveries were only possible because of the high statistical power of large-sample studies. They are also relatively recent efforts. Now, at the outset of animal neuroimaging data-sharing initiatives, the aggregation of high volumes of data stands to enable us to address a range of scientific questions of high translational relevance (Zerbi et al., 2021). Fostering the growth of these resources will be critical in the coming years. Although we can learn a lot from how large human neuroimaging datasets have been amassed and are managed, similar efforts in the animal neuroimaging field require some special considerations. For example, we acquire a wider variety of data, the burden of doing so is substantially greater and on the shoulders of trainees, or early career scientists.

Despite the clear benefits of large datasets, we must remember that for animal neuroimaging these come at a high cost. It is a privilege to work with animals; they enable us to conduct investigations that would never be doable in humans. Yet, we cannot forget that the lives of the animals we use are forfeited in our pursuit of knowledge. We have an ethical obligation to limit their number through efforts to replace animals wherever possible with alternatives and to refine our experiments such that fewer animals are needed to answer our questions. We must observe the ethical guidelines laid out by the 3Rs principle.

As in large-scale human neuroimaging efforts, in animal neuroimaging, we are practically limited to aggregative initiatives. Although the community is equipped with computational methods to harmonize data acquired across sites, aggregative efforts (by definition) are only practical for simple, mainstream, and longstanding paradigms. Not the more creative and unique efforts that are arguably the true strength of animal neuroimaging efforts. Hence, using sample size as the sole metric by which we establish the impact and validity of a study greatly limits the innovative power and pioneering spirit of animal neuroimaging research. This is particularly valid for research questions aimed at addressing mechanistic hypotheses, which is one of the most significant contributions that animal neuroimaging stands to produce for science. To test these multiscale hypotheses, “deep” datasets with multimodal sources and manipulations (not available in human research) have a much higher explanatory impact relative to “wide” (and shallow) datasets composed of large numbers of subjects but use a single technique.

“What N is N-ough?” is an open question for animal neuroimaging with no one-size-fits-all solution. Rather, sample size must be considered alongside the specific research question being asked. Large and small datasets come with their respective advantages and challenges. An equally meaningful (and more insightful) question is: “How can we help the community to make more informed decisions on the number of animals needed for an investigation?”. To this aim, immediate actions can be promptly taken. Action item number one is publishing effect sizes together with *p* values. Like other research fields, information on effect sizes is often missing in animal neuroimaging and is critical for calculating sample size. Action item number two is publishing null or negative results and replication studies. Decisions on sample size are based on shared findings and publication biases skew our ability to make these estimates. Both these items stand to have a big impact on guiding our determination of adequate and responsible sample sizes as we move forward.

## Synthesis

Reviewing Editor: Sam Golden, The University of Washington Decisions are customarily a result of the Reviewing Editor and the peer reviewers coming together and discussing their recommendations until a consensus is reached. When revisions are invited, a fact-based synthesis statement explaining their decision and outlining what is needed to prepare a revision will be listed below. The following reviewer(s) agreed to reveal their identity: Teresa Gunn.

Dear Dr. Mandino and Team,

Your manuscript has been read by two experts in the field and favorably reviewed. Here I provide a synthesis of their main comments, followed by Reviewer Comments themselves. Pending editorial review of a point-by-point rebuttal and revised manuscript, a final decision will be provided.

The comments from two reviewers present a mix of appreciation and fair critique. Reviewer 1 commends the article for its well-written discussion on the pros and cons of adopting sample sizes in animal studies comparable to those recommended for human research, highlighting its utility for grant and paper reviewers in assessing study adequacy. Reviewer 2, however, raises several addressable concerns, notably the broad use of the term "neuroimaging" when the article mainly addresses fMRI studies, suggesting a need for specificity to avoid confusion. Furthermore, Reviewer 2 criticizes the use of subjective terms, the comparison of sample sizes without adequate context, and the assumption that the goal of all animal neuroimaging studies is to generalize findings to humans. These critiques suggest a need for more clear terminology and assumptions to ensure their arguments are accurately and clearly presented to the readership. Overall, the reviewers were enthusiastic about the potential for this perspective to be well received by the field.

Reviewer 1:

This is a well-written and thoughtful article identifying and addressing the "pros and cons" of increasing sample sizes in animal neuroimaging studies to n=1000, on par with that currently recommended for human studies. It will be of interest to those in the field and useful to (for example) reviewers of grants and papers with animal neuroimaging studies, to help them assess whether study n's are appropriate and adequate.

Reviewer 2:

"Neuroimaging" is too broad of a term to be used in this context as the article primarily deals with the modality of fMRI. This is especially the case for a broad journal like eNeuro as there are many different types of imaging modalities that can fall under the category of "neuroimaging" and for some, the arguments presented in this article are irrelevant. I suggest making the title and the rest of the article more explicit in terms of what specific type of neuroimaging studies (e.g., fMRI-based) the article refers to. Otherwise, it may confuse the readership.

Line 19: I suggest using less subjective terms (e.g., "Saddest") when presenting an argument. This is also seen in line 100 ("shameful"). It can confuse the reader and lead to a misinterpretation of the argument.

Line 50: What exactly is "very low"? Compared to what? N=15 is not a low sample size for rat studies. It is of course low when compared to 1000s of humans, so I would consider rephrasing this sentence accordingly.

Line 81: This is not the goal of all animal neuroimaging studies. Not all animal neuroimaging studies are preoccupied with generalizing their findings to humans. For example, using PET, n=1 is sufficient to detect the expression and location of an exogenous transgene in the brain. This is directly applicable to gene therapy applications (e.g., personalized medicine). So, for this argument, the authors should consider being more specific in their statements.

Line 109: what exactly is meant by using the term "epigenetically" in parentheses? Is the author claiming that these mice are epigenetically identical? This is confusing to me and the authors should consider rephrasing the sentence or removing this term.

## References

[B3] Bethlehem RAI, et al. (2022) Brain charts for the human lifespan. Nature 604:525–533. 10.1038/s41586-022-04554-y 35388223 PMC9021021

[B4] Bonapersona V, Hoijtink H, RELACS Consortium, Sarabdjitsingh RA, Joëls M (2021) Increasing the statistical power of animal experiments with historical control data. Nat Neurosci 24:470–477. 10.1038/s41593-020-00792-3 33603229

[B5] Buckser RR (2021) A hitchhiker’s guide to OpenNeuro: secondary analysis on the web’s largest repository of open neuroimaging data.

[B6] Carp J (2012) The secret lives of experiments: methods reporting in the fMRI literature. Neuroimage 63:289–300. 10.1016/j.neuroimage.2012.07.004 22796459

[B7] Cipriani A, et al. (2018) Comparative efficacy and acceptability of 21 antidepressant drugs for the acute treatment of adults with major depressive disorder: a systematic review and network meta-analysis. Lancet 391:1357–1366. 10.1016/S0140-6736(17)32802-7 29477251 PMC5889788

[B8] Desrosiers-Gregoire G, Devenyi GA, Grandjean J (2022) Rodent automated bold improvement of EPI sequences (RABIES): a standardized image processing and data quality platform for rodent fMRI. bioRxiv. 10.1038/s41467-024-50826-8 PMC11306392 39112455

[B9] Di Martino A, et al. (2014) The autism brain imaging data exchange: towards a large-scale evaluation of the intrinsic brain architecture in autism. Mol Psychiatry 19:659–667. 10.1038/mp.2013.78 23774715 PMC4162310

[B10] Freund J, et al. (2013) Emergence of individuality in genetically identical mice. Science 340:756–759. 10.1126/science.1235294 23661762

[B11] Goldberg TE, Lee S, Devanand DP, Schneider LS (2023) Comparison of relative change with effect size metrics in Alzheimer'’s disease clinical trials. J Neurol Neurosurg Psychiatry 951:2–7. 10.1136/jnnp-2023-331941 PMC11299058 37979967

[B12] Gordon EM, et al. (2023) A somato-cognitive action network alternates with effector regions in motor cortex. Nature 617:351–359. 10.1038/s41586-023-05964-2 37076628 PMC10172144

[B13] Gorgolewski KJ, et al. (2016) The brain imaging data structure, a format for organizing and describing outputs of neuroimaging experiments. Sci Data 3:160044. 10.1038/sdata.2016.44 27326542 PMC4978148

[B14] Grandjean J, et al. (2020) Common functional networks in the mouse brain revealed by multi-centre resting-state fMRI analysis. Neuroimage 205:116278. 10.1016/j.neuroimage.2019.116278 31614221 PMC7116112

[B15] Grandjean J, et al. (2023) A consensus protocol for functional connectivity analysis in the rat brain. Nat Neurosci 26:673–681. 10.1038/s41593-023-01286-8 36973511 PMC10493189

[B16] Hooijmans CR, Leenaars M, Ritskes-Hoitinga M (2010) A gold standard publication checklist to improve the quality of animal studies, to fully integrate the three Rs, and to make systematic reviews more feasible. Altern Lab Anim 38:167–182. 10.1177/026119291003800208 20507187

[B1] International Brain Laboratory (2017) An international laboratory for systems and computational neuroscience. Neuron 96:1213–1218. 10.1016/j.neuron.2017.12.013 29268092 PMC5752703

[B17] Ioannidis JPA (2005) Why most published research findings are false. PLoS Med 2:e124. 10.1371/journal.pmed.0020124 16060722 PMC1182327

[B18] Jullienne A, Trinh MV, Obenaus A (2022) Neuroimaging of mouse models of Alzheimer’s disease. Biomedicines 10:305. 10.3390/biomedicines10020305 35203515 PMC8869427

[B19] Lake EMR, et al. (2020) Simultaneous cortex-wide fluorescence Ca2+ imaging and whole-brain fMRI. Nat Methods 17:1262–1271. 10.1038/s41592-020-00984-6 33139894 PMC7704940

[B20] Lein ES, et al. (2007) Genome-wide atlas of gene expression in the adult mouse brain. Nature 445:168–176. 10.1038/nature05453 17151600

[B21] Mandino F, et al. (2019) Animal functional magnetic resonance imaging: trends and path toward standardization. Front Neuroinform 13:78. 10.3389/fninf.2019.00078 32038217 PMC6987455

[B22] Mandino F, et al. (2022) The lateral entorhinal cortex is a hub for local and global dysfunction in early Alzheimer’s disease states. J Cereb Blood Flow Metab 42:1616–1631. 10.1177/0271678X221082016 35466772 PMC9441719

[B23] Mandino F, Vujic S, Grandjean J, Lake EMR (2024) Where do we stand on fMRI in awake mice? Cereb Cortex 34:bhad478. 10.1093/cercor/bhad478 38100331 PMC10793583

[B24] Marek S, et al. (2022) Publisher correction: reproducible brain-wide association studies require thousands of individuals. Nature 605:E11. 10.1038/s41586-022-04692-3 35534626 PMC9132768

[B25] Markiewicz CJ, et al. (2021) The OpenNeuro resource for sharing of neuroscience data. Elife 10:e71774. 10.7554/eLife.71774 34658334 PMC8550750

[B26] Maxwell O, Udoka Chinedu I, Charles Ifeanyi A (2019) Numbers in life: a statistical genetic approach. Guigoz Sci Rev 5:142–149. 10.32861/sr.57.142.149

[B27] Mintun MA, Wessels AM, Sims JR (2021) Donanemab in early Alzheimer’s disease. Reply. N Engl J Med 384:1691–1704. 10.1056/NEJMoa2100708 33720637

[B28] Mohammad F, et al. (2016) Concordance and incongruence in preclinical anxiety models: systematic review and meta-analyses. Neurosci Biobehav Rev 68:504–529. 10.1016/j.neubiorev.2016.04.011 27328783

[B29] Oddo S, et al. (2003) Triple-transgenic model of Alzheimer’s disease with plaques and tangles: intracellular Abeta and synaptic dysfunction. Neuron 39:409–421. 10.1016/S0896-6273(03)00434-3 12895417

[B30] Oh SW, et al. (2014) A mesoscale connectome of the mouse brain. Nature 508:207–214. 10.1038/nature13186 24695228 PMC5102064

[B2] {PRIMatE Data Exchange (PRIME-DE) Global Collaboration Workshop and Consortium (2020) Accelerating the evolution of nonhuman primate neuroimaging. Neuron 105:600–603. 10.1016/j.neuron.2019.12.023 32078795 PMC7610430

[B31] Rocchi F, et al. (2022) Increased fMRI connectivity upon chemogenetic inhibition of the mouse prefrontal cortex. Nat Commun 13:1056. 10.1038/s41467-022-28591-3 35217677 PMC8881459

[B32] Russell WMS (1995) The development of the three Rs concept. Altern Lab Anim 23:298–304. 10.1177/026119299502300306 11656565

[B33] Van Essen DC, et al. (2012) The Human Connectome Project: a data acquisition perspective. Neuroimage 62:2222–2231. 10.1016/j.neuroimage.2012.02.018 22366334 PMC3606888

[B34] Van Essen DC, et al. (2013) The WU-minn human connectome project: an overview. Neuroimage 80:62–79. 10.1016/j.neuroimage.2013.05.041 23684880 PMC3724347

[B35] Williams R (2010) The human connectome: just another ‘ome? Lancet Neurol 9:238–239. 10.1016/S1474-4422(10)70046-6 20170838

[B36] Xu T, et al. (2020) Cross-species functional alignment reveals evolutionary hierarchy within the connectome. Neuroimage 223:117346. 10.1016/j.neuroimage.2020.117346 32916286 PMC7871099

[B37] Yokoyama M, Kobayashi H, Tatsumi L, Tomita T (2022) Mouse models of Alzheimer’s disease. Front Mol Neurosci 15:912995. 10.3389/fnmol.2022.912995 35799899 PMC9254908

[B38] Zerbi V, et al. (2021) Brain mapping across 16 autism mouse models reveals a spectrum of functional connectivity subtypes. Mol Psychiatry 26:7610–7620. 10.1038/s41380-021-01245-4 34381171 PMC8873017

